# The involvement of hyperhomocysteinemia in the development of characterized depressive disorder in children and adolescents

**DOI:** 10.1192/j.eurpsy.2023.633

**Published:** 2023-07-19

**Authors:** Z. Elmaataoui, H. BELHADGA, H. KISRA

**Affiliations:** HOSPITAL AR-RAZI OF SALE, SALE, Morocco

## Abstract

**Introduction:**

Elevated blood levels of homocysteine have been associated with several psychiatric and neurodegenerative disorders such as schizophrenic disorders, Alzheimer’s disease, Parkinson’s disease and depression. The hypothesis is that genetic and environmental factors elevate homocysteine levels, which causes vascular diseases of the brain, and/or changes in neurotransmitters, which cause various mental disorders.

**Objectives:**

The objective of our work is to discuss the association between hyperhomocysteinemia and the characterized depressive disorder

**Methods:**

we conducted our study through the discussion of a clinical vignette

**Results:**

We report here a case of hyperhomocysteinemia with vitamin B 12 deficiency in a 16-year-old female patient who presented with a characterized depressive disorder.

She was initially treated with a selective serotonin inhibitor combined with parenteral injections of vitamin B12. The patient’s clinical condition improved after the first week. The discussion will attempt to clarify the role of vitamin therapy in the improvement of the patient’s depressive symptoms and its relationship with hyperhomocysteinemia.

We report here a case of hyperhomocysteinemia with vitamin B 12 deficiency in a 16-year-old female patient who presented with a characterized depressive disorder.She was initially treated with a selective serotonin inhibitor combined with parenteral injections of vitamin B12. The patient’s clinical condition improved after the first week. The discussion will attempt to clarify the role of vitamin therapy in the improvement of the patient’s depressive symptoms and its relationship with hyperhomocysteinemia.

**Conclusions:**

Statistical data, physiological and genetic aspects seem to point to the involvement of hyperhomocysteinemia in the development of characterized depressive disorder. However, the results remain variable, even contradictory, and several confounding factors must be considered in these studies: ethnic, geographical, cultural (in terms of diet) and age factors are all elements that seem to intervene and that do not always make it possible to know whether hyperhomocysteinemia is a direct cause of depression or the consequence of mechanisms linked to folate and B12 deficiencies.

**Disclosure of Interest:**

None Declared

